# A pragmatic workflow for handling missing PROM data in registry studies: a methodological scoping review and simulated worked example

**DOI:** 10.1186/s12874-026-03058-x

**Published:** 2026-10-19

**Authors:** Johan Eriksson

**Affiliations:** Department of Diagnostics and Intervention/Surgery, Umeå University, Umeå, Sweden

**Keywords:** PROM, Registries, Missing data, Non-response, Inverse probability weighting, MNAR, Sensitivity analysis, Delta adjustment, Tipping point

## Abstract

**Background:**

Patient-reported outcome measures (PROMs) are increasingly integrated into clinical quality registries. Registry-based PROM data frequently exhibit structural non-response, including patients who never return PROMs (“never-responders”), and longitudinal attrition, making missing-not-at-random (MNAR) mechanisms plausible. Although MNAR sensitivity methods such as delta adjustment and tipping-point analysis are methodologically well-described, their uptake in registry-PROM practice is unknown.

**Methods:**

A structured search of PubMed (Nordic-specific terms), PubMed (global terms), Embase and CINAHL was conducted (2018–2024), yielding 425 records. After deduplication and screening, 61 registry-based observational PROM studies with explicit missing-data methods discussion were included as the primary analytic sample (17 Nordic, 44 global). The audit was conducted as a methodological scoping review and reported in accordance with the PRISMA extension for scoping reviews (PRISMA-ScR). A three-step pragmatic workflow is proposed and demonstrated: (1) non-response diagnostics separating never-responders from dropout, with standardized mean differences (SMD); (2) inverse probability weighting (IPW) as a default primary analysis strategy; and (3) MNAR sensitivity via delta adjustment with explicit tipping-point identification and a simple robustness classification. The workflow is demonstrated in a simulated registry-like cohort (*N* = 8,000).

**Results:**

In the primary analytic sample (*n* = 61), 24 studies (39%) stated no missing-data strategy. MNAR sensitivity analysis was reported in 18/61 (30%), but formal MNAR models were used in only 5/61 (8%). Critically, tipping-point analysis was not reported in any of the 61 included studies (0%); delta adjustment was reported in only 1/61 (2%). Never-responders were distinguished from dropout in only 17/61 (28%). In the simulated cohort, non-response was substantial and selective. In the primary scenario, the complete-case estimate was 1.21 PROM points (95% CI: 0.69–1.72), the single-stage IPW estimate was 1.22 (95% CI: 0.69–1.74), and the two-stage IPW estimate was 1.23 (95% CI: 0.67–1.79). The tipping point was δ* = 7.2 points (classified as sensitive under the illustrative thresholds). In the secondary scenario, the corresponding tipping point was δ* = 30.5 points (classified as stable).

**Conclusions:**

Tipping-point analysis was not reported in the accessible methods or supplementary material of any of the 61 included registry-PROM studies; delta adjustment was reported in only one study as a sensitivity check. The five formal MNAR analyses identified used methods that typically require specialist expertise and stronger modeling assumptions than are routinely feasible in many registry settings. A pragmatic workflow using IPW and tipping-point analysis can close this gap without specialist infrastructure.

**Supplementary Information:**

The online version contains supplementary material available at https://doi.org/10.1186/s12874-026-03058-x.

## Background

Patient-reported outcome measures (PROMs) complement clinical endpoints by capturing symptoms, function, and health-related quality of life from the patient’s perspective. Their integration into clinical quality registries has expanded rapidly in the Nordic countries and beyond, enabling large-scale observational analyses at low marginal cost [1]. However, the inferential value of registry-based PROMs is threatened by two structurally distinct forms of missing data.

First, a proportion of patients never engage with PROM collection, so-called never-responders, whose non-participation is typically associated with older age, higher comorbidity burden, lower socioeconomic status, and more severe disease [2, 3]. Second, among patients who respond at baseline, a further proportion drops out at subsequent timepoints, often because deteriorating health reduces capacity or motivation to respond. This second mechanism is of particular methodological concern: the missing PROM value may depend on the unobserved outcome even after conditioning on observed covariates, constituting a missing-not-at-random (MNAR) mechanism [4].

Registry-PROM studies face constraints that distinguish them from randomized trial settings: no randomization, limited psychosocial predictors of response, large sample sizes with potentially small but clinically meaningful biases, and missingness patterns embedded in healthcare trajectories. Methodological guidance specifically tailored to this setting remains limited.

MNAR sensitivity methods, including delta adjustment and tipping-point analysis, are methodologically well-described [4–6]. The tipping-point framework is particularly appealing for registry practice: it answers a clinically interpretable question (“how much worse would missing patients need to be for our conclusion to change?”) without committing to an unverifiable parametric model [5, 7]. Yet whether these methods are used in registry-PROM practice remains unknown.

To characterize the gap between methodological availability and practical uptake, a structured search and full-text extraction of Nordic registry-based PROM studies (2018–2024) was conducted. The focus of this paper is specifically on registry-based PROM analyses evaluating treatment effects in observational settings, rather than descriptive monitoring or psychometric applications. The search was anchored in the Nordic literature using registry-specific nomenclature (PubMed), supplemented by global searches in Embase, CINAHL, and PubMed using design-based terms. Together these sources provide a broad cross-national characterization of registry-PROM reporting practice. The audit findings motivate the three-step workflow proposed here.

## Methods

### Structured search and full-text audit

#### Search strategy and study selection

Searches were conducted in three databases (January 2018–December 2024). In PubMed, two complementary strategies were applied: a Nordic-specific search using registry nomenclature (e.g., “NKBC”, “Swespine”, “DANBIO”, “NORspine”, “GLA: D”, “Norwegian”, “Danish”, “Finnish”) combined with PROM terms (“patient-reported outcome*”, “PROM”, “HRQoL”) and missingness terms (“missing”, “non-response”, “attrition”), yielding 39 records; and a design-based search using the Registries[MeSH] term combined with the same PROM and missingness terms, yielding 41 records. In Embase (embase.com) and CINAHL (EBSCO), design-based registry terms (“clinical registry”, “quality registry”, “national registry”, “routinely collected”, “registry-based”) were combined with the same PROM and missingness terms, yielding 251 and 94 records respectively. After deduplication (~ 28 duplicates removed), 397 unique records were screened. The audit was conducted as a methodological scoping review of reporting and analytical practices in registry-based observational PROM studies and is reported in accordance with the PRISMA extension for scoping reviews (PRISMA-ScR) [17], informed by the JBI scoping-review methodology [18]; the RECORD statement [8] is used as a domain-specific reporting reference for the registry-based studies under audit. No review protocol was registered.

Three sequential levels of classification were applied to all retrieved records. First, records were examined to identify registry/routine-data observational PROM studies, defined as observational studies in which a clinical registry or routine healthcare data constituted the primary data source and at least one PROM was included as a study outcome. Randomized trials, validation-only studies, feasibility and pilot studies, protocols, reviews, qualitative studies, and cross-sectional surveys without registry linkage were excluded at this level. Second, a primary analytic sample was defined as studies with explicit missing-data handling discussion in the methods or statistical analysis section, i.e., studies that described a specific analytic approach to missing data, a missingness mechanism (missing at random [MAR], missing not at random [MNAR], or missing completely at random [MCAR]), or a comparison of responders and non-responders as part of their statistical methods. Studies that only reported a response rate or noted missing data in passing were not included. Third, full texts were retrieved; records not obtainable (*n* = 1 not found, *n* = 3 behind paywall) were excluded. This process yielded 61 included studies: 17 from the Nordic PubMed search and 44 from the global searches.

#### Data extraction and coding

Full-text methods sections were screened for five prespecified items: (1) whether a missing-data strategy was explicitly stated; (2) whether a responder vs. non-responder comparison was conducted; (3) whether never-responders were distinguished from follow-up dropout; (4) whether any MNAR sensitivity was reported; and (5) whether delta-adjustment or tipping-point analysis was reported. Items absent from the methods section were coded “not stated” rather than “not done”.

MNAR sensitivity was coded at two levels. Informal MNAR analysis: text stating or implying that data were not MCAR or probably MNAR (e.g., “data were not missing at random”, MCAR plausibility checks via early vs. late responder comparisons), without a formal sensitivity model. Formal MNAR analysis: an explicit MNAR model or prespecified sensitivity framework, such as a selection model, shared-parameter/joint model, pattern-mixture model, or a structured MNAR classification using predefined criteria (e.g., minimal important difference (MID) thresholds).

Delta-adjustment or tipping-point analysis was coded as present only if described in the methods or statistical analysis section, or in supplementary methods. It was coded as not reported if not mentioned in any of these locations.

The complete extraction is provided in Supplementary Table S1.

### Proposed three-step workflow

**Fig. 1:**
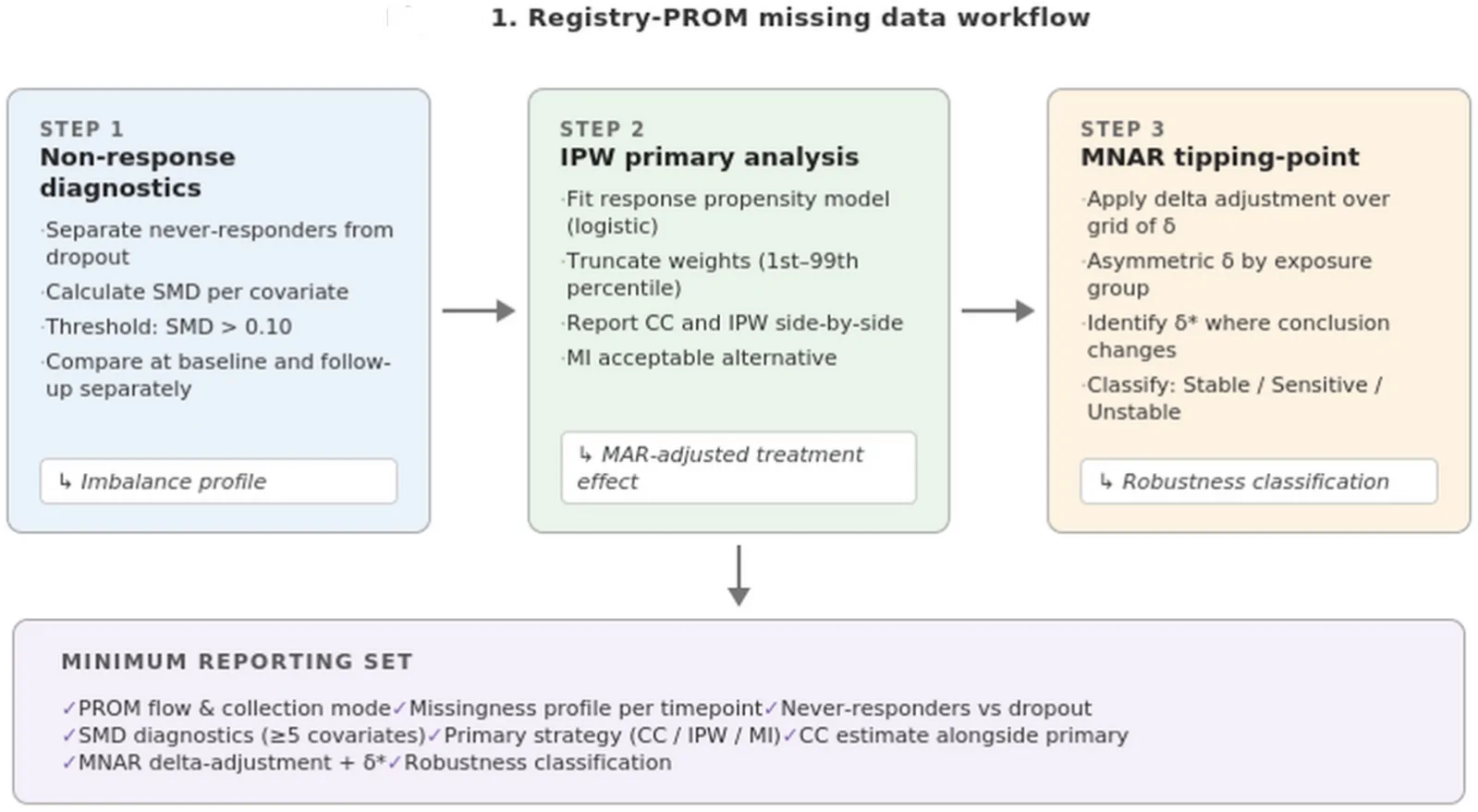
Three-step workflow for handling missing PROM data in registry-based studies. Step 1 produces a covariate imbalance profile (SMD love plot) that informs propensity model specification in Step 2. Step 2 yields an IPW-adjusted treatment effect under missing-at-random (MAR), reported alongside the complete-case estimate. Step 3 applies asymmetric delta adjustment across a grid of δ values to identify the tipping point δ at which the primary conclusion would be reversed, with results classified as Stable (δ* > 10), Sensitive (δ* 5–10), or Unstable (δ* < 5). Outputs from all three steps map directly to the Minimum Reporting Set (Table 1). The δ thresholds shown are illustrative only and should be adapted to the minimally important difference (MID) of the instrument in use. SMD = standardized mean difference; IPW = inverse probability weighting; MAR = missing at random; MNAR = missing not at random; CC = complete case.

The proposed workflow is summarized in Fig. 1, and its minimum reporting components are listed in Table 1.

**Table 1:** Minimum reporting set for registry-based PROM studies with missing data

Domain	Minimum requirement	Rationale
PROM flow	Collection timepoints; mode of data collection (paper/electronic/mixed); N invited per timepoint	Enables identification of structural missingness patterns
Missingness profile	Proportion missing per timepoint; never-responders distinguished from follow-up dropout	Missingness type determines appropriate analytic strategy
Non-response diagnostics	Responder vs. non-responder comparison on ≥ 5 prespecified baseline covariates with SMD (the threshold of five covariates ensures coverage of key clinical and sociodemographic domains — age, comorbidity, disease severity, socioeconomic factors, and treatment)	Quantifies potential for selection bias; SMD preferred over p-values
Primary strategy	Method (CC/IPW/MI); model specification and predictors; weight diagnostics if IPW	Reproducibility and critical appraisal of MAR assumption
Results	CC and primary strategy estimates reported side-by-side	Transparent comparison; highlights sensitivity to strategy choice
MNAR sensitivity	At least one delta/tipping-point analysis: range of δ tested + δ* where conclusion changes	Quantifies how severe MNAR would need to be to change inference
Robustness classification	Stable (δ*>10) / Sensitive (δ* 5–10) / Unstable (δ*<5) — thresholds adapted to instrument scale	Clinically interpretable summary of MNAR sensitivity

CC = complete-case analysis; IPW = inverse probability weighting; MI = multiple imputation; SMD = standardized mean difference. Robustness classification thresholds are indicative and should be adapted to the scale and minimally important difference of the PROM instrument used

#### Step 1 – Non-response diagnostics

A structural distinction is recommended between never-responders, patients providing no PROM data at any timepoint, and dropout, defined as non-response at a follow-up occasion among baseline responders. Non-response diagnostics compare responders and non-responders on prespecified baseline covariates using standardized mean differences (SMD) [9]. SMD is preferred over p-values, which conflate effect size with sample size and are uninformative for characterizing selection. An SMD exceeding 0.10 indicates potentially meaningful imbalance, following conventional thresholds used in propensity score and covariate balance assessment [9]. Comparisons should be performed separately for baseline non-response and follow-up dropout. These diagnostics may be presented as an SMD love plot to visualize covariate imbalance.

#### Step 2 – Primary analysis strategy (IPW-first)

Inverse probability weighting (IPW) is recommended as the default primary analysis for registry-based PROM studies [10]. IPW reweights observed cases by the inverse of their estimated response propensity, up-weighting patients whose characteristics make them less likely to respond. A response propensity model is fitted using logistic regression among baseline responders, with follow-up response as the outcome. Each observed respondent’s predicted probability of response is estimated from this model, and the inverse of that probability is used as an analytic weight. Weight truncation at the 1st and 99th percentiles is recommended to limit extreme weight influence. Both complete-case (CC) and IPW estimates should be reported side-by-side to enable comparison. IPW estimates should be accompanied by robust (sandwich/Huber-White) standard errors for weighted analyses, which account for variability in the estimated weights [19]; the bootstrap or stacked estimating equations can be used when uncertainty in the weight model is to be propagated more fully. Naive model-based standard errors treat weights as fixed and known, underestimating variance.

Multiple imputation (MI) under a MAR assumption is an acceptable alternative when a richer covariate structure is available [5, 6]; MI and IPW can serve as mutual sensitivity analyses.

When both structural baseline non-response and follow-up dropout are present, the inverse-probability weight can be formed as the product of two stage-specific response probabilities: a baseline-response model fitted over the full cohort using registry covariates (baseline PROM is undefined for non-responders), and a follow-up-response model fitted among baseline responders, conditioning additionally on the observed baseline PROM. The combined two-stage weight, w = 1 / [P(baseline response) × P(follow-up response)], targets the full eligible population under a sequential conditional-MAR assumption at each stage. Because never-response is plausibly MNAR, this baseline step corrects only the component explainable by observed covariates; the residual MNAR risk is precisely what the Step 3 tipping-point analysis probes.

#### Step 3 – MNAR sensitivity: delta adjustment and tipping point

The tipping-point approach answers a clinically interpretable question: how severely would the MAR assumption need to be violated before the substantive conclusion changes [5, 8]? Missing PROM values are assumed to be, on average, δ points lower (worse) than predicted under MAR. The analysis is repeated across a grid of δ values, and the tipping point δ* is the value at which the estimated treatment effect crosses zero or a prespecified clinical threshold. To reflect asymmetric MNAR mechanisms, the delta adjustment can be applied differentially by exposure group. Rather than applying fixed numerical thresholds, δ* should be interpreted relative to the minimally important difference (MID) or meaningful score difference (MSD) of the PROM instrument in use, ideally derived from anchor-based methods [11]. For illustrative purposes in the worked example below, a classification scheme of stable (δ* > 10 points), sensitive (δ* 5–10 points), and unstable (δ* < 5 points) is applied; these thresholds correspond roughly to effect sizes of d > 1.25, d 0.6–1.25, and d < 0.6 respectively, given the simulated residual SD of ~ 8 points. Researchers should adapt these boundaries to the established MID of their instrument [7].

### Simulated worked example

#### Cohort design and missingness mechanisms

A registry-like cohort was simulated (*N* = 8,000) with covariates reflecting a typical Nordic register: age (mean 62 years, SD 10), disease stage (three levels: 55/30/15%), comorbidity count (Poisson, λ = 1.2), socioeconomic status (SES) proxy (35% low SES), and a binary treatment indicator (45% treated). A continuous PROM score (0–100, higher = better) was generated at baseline and 12-month follow-up, with a true treatment effect of + 0.8 PROM points in the primary scenario. This effect size is intentionally sub-clinical (Cohen’s d ≈ 0.10 given the simulated residual SD of ~ 8 points) and was chosen to illustrate tipping-point mechanics under conditions of genuine fragility; smaller true effects will inherently yield lower tipping points. To demonstrate workflow behavior under a clinically meaningful treatment effect, a secondary scenario with a true effect of + 4.0 PROM points (Cohen’s d ≈ 0.50) is reported alongside. PROM scores were bounded to the instrument range [0, 100] by clamping (values below 0 set to 0 and values above 100 set to 100), applied both at generation and after delta adjustment.

Three missingness components were simulated: (1) never-responders whose probability of structural non-response depended on age, SES, comorbidity, and stage (≈ 37% of the cohort); (2) sporadic baseline non-response among non-never-responders; and (3) MNAR dropout at follow-up, where dropout probability depended on both observed covariates and the unobserved follow-up PROM value, with a differential component by treatment group. For the tipping-point analysis, an asymmetric delta specification was used: missing outcomes in the treatment group were shifted by δ points; control-group missing outcomes by δ/3 points. The ratio of δ/3 for the control group is illustrative; in applied analyses, the relative magnitude of differential MNAR between groups should be informed by clinical context, expected treatment toxicity, or plausible non-response mechanisms rather than a default ratio. All analyses were conducted in R (version 4.5.1). The full simulation and analysis script is provided as a supplementary file.

## Results

### Full-text audit

Of 425 records identified, 397 remained after deduplication; 82 were retrieved for full-text review (4 not retrievable), and 61 met all inclusion criteria and formed the primary analytic sample (see PRISMA flow diagram, Supplementary Figure S1; full search terms and per-database counts are reported in the Methods): 17 from the Nordic PubMed search and 44 from the global searches. The complete extraction is provided in Supplementary Table S1. Confidence intervals for key proportions are Clopper-Pearson exact 95% CI.

Among the 61 primary analytic sample studies, 24 (39%, 95% CI: 27–52%) stated no missing-data strategy, relying implicitly on complete-case analysis. Thirty-three studies (54%, 95% CI: 41–67%) reported a responder vs. non-responder comparison, but standardized mean differences were rarely used; the majority used unadjusted statistical tests or qualitative descriptions. Only 17 studies (28%, 95% CI: 17–41%) distinguished never-responders from follow-up dropout. The range of overall missingness was not consistently reported in a comparable format; where stated, proportions with missing follow-up PROM data ranged from approximately 10% to over 80%.

MNAR sensitivity analysis, formal or informal, was reported in 18/61 studies (30%). Informal MNAR handling (13 studies) consisted of statements that data were probably not MCAR, or of MCAR plausibility checks via early vs. late responder comparisons, without a formal sensitivity analysis. Five studies used formal MNAR models: a shared-parameter joint model accounting for death-related dropout [12], two selection models [13, 14], an MNAR classification based on minimal important difference thresholds [15], and one study that applied delta adjustment as a sensitivity check [16]. These methods require specialist statistical expertise and stronger modeling assumptions than are routinely feasible in many registry settings.

Tipping-point analysis was not reported in the accessible methods or supplementary material of any of the 61 included studies (0%). Delta adjustment was identified in one study (Hoskins et al. 2024 [16], 1.6%), applied as a sensitivity scenario rather than a primary analytic strategy. No study reported use of a structured tipping-point framework to classify result robustness.

**Table 2:** Full-text audit of missing data reporting in registry-based PROM studies (2018–2024; *n* = 61)

Reporting item	Total sample (*n* = 61)	Notes
Missing data strategy explicitly stated	37/61 (61%)	CC, MI, IPW, model-based, or single imputation
No strategy stated (implicit CC)	24/61 (39%)	Missing-data approach not described in methods/stats section
Responder vs. non-responder comparison	33/61 (54%)	Usually unadjusted tests; SMD used in < 2 studies
Never-responders distinguished from follow-up dropout	17/61 (28%)	Most studies treat all non-response as one category
MNAR sensitivity analysis reported^a^	18/61 (30%)	Includes informal MNAR (13/61) and formal MNAR models (5/61)
No MNAR sensitivity analysis reported	43/61 (70%)	
Formal MNAR model^b^	5/61 (8%)	Joint/shared-parameter model; selection model; MID-based classification
Delta adjustment or tipping-point analysis	**1/61 (1.6%)**	Tipping-point analysis not reported in any of the 61 included studies; delta adjustment reported in 1/61 (Hoskins 2024, as sensitivity check)

Full-text audit of missing-data reporting in registry-based PROM studies (2018–2024; *n* = 61)

CC = complete-case analysis; MI = multiple imputation; IPW = inverse probability weighting; MNAR = missing not at random; MID = minimally important difference; SMD = standardized mean difference

ᵃ Informal MNAR: statement that data were probably not MCAR, or plausibility checks for non-random missingness, without a formal sensitivity model

ᵇ Formal MNAR: explicit MNAR model or prespecified sensitivity framework, including shared-parameter/joint models, selection models, MID-based classification, or delta adjustment

### Simulated worked example: missingness profile and diagnostics

Of 8,000 simulated patients, 4,753 (59%) provided baseline PROM data. Never-responders numbered 2,928 (37%), with a further 319 (4%) having sporadic baseline non-response. Among baseline responders, 924 (19%) dropped out at 12-month follow-up, leaving 3,829 (48%) with complete PROM data.

**Fig. 2:**
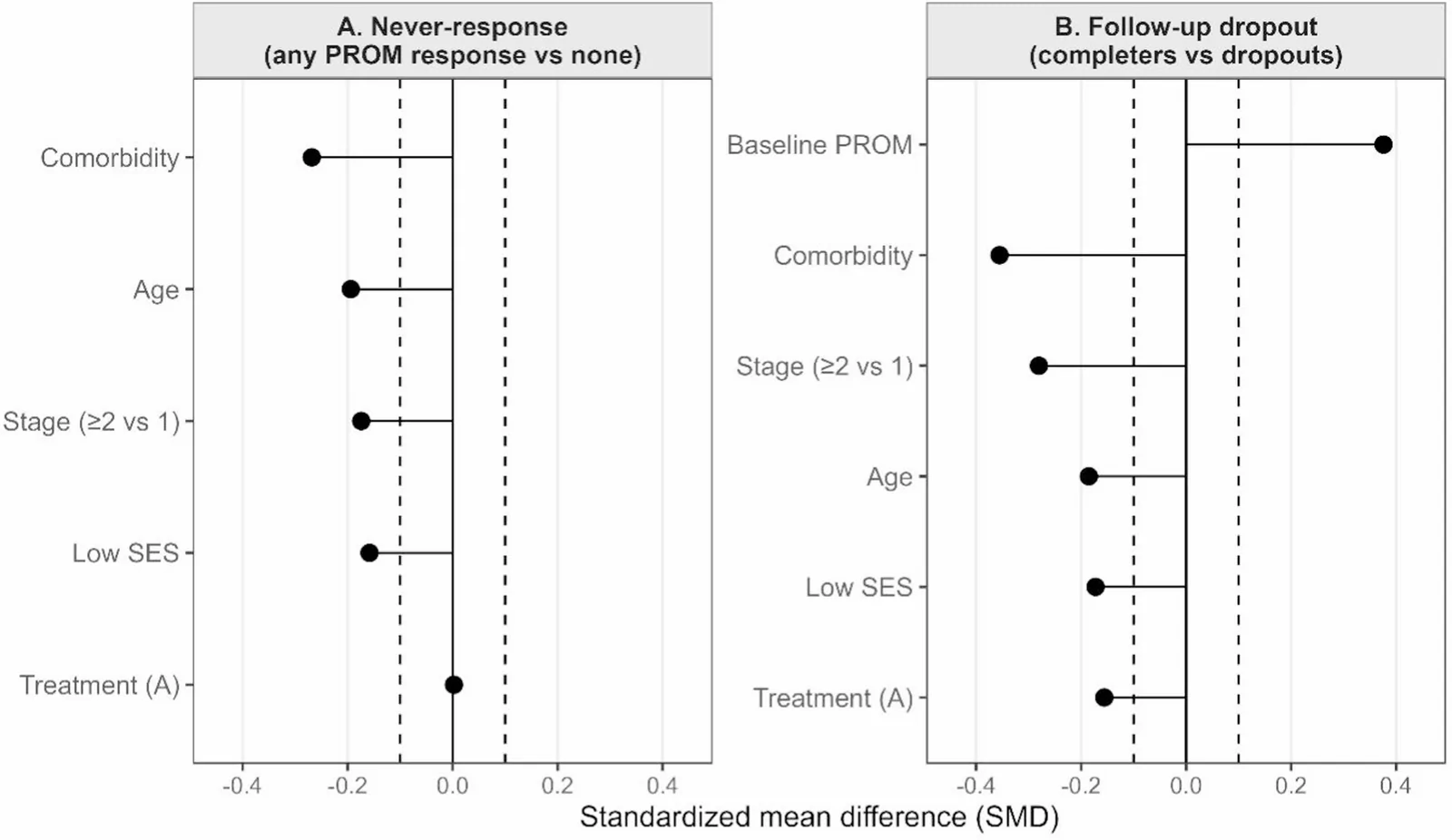
SMD love plot. Non-response diagnostics presented separately for the two missingness mechanisms in the simulated cohort. Panel A: standardized mean differences (SMD) between patients with any PROM response and never-responders. Panel B: SMD between completers and follow-up dropouts among baseline responders. Negative SMD values indicate lower values or lower prevalence in the responding/completing group relative to the non-responding/dropout group. Dashed vertical lines indicate SMD = ± 0.10. Simulated cohort, *N* = 8000

Non-response diagnostics were computed separately for the two mechanisms (Fig. 2). For never-response (any PROM response versus none), imbalance was driven by comorbidity (SMD − 0.27), age (− 0.19), disease stage (− 0.17) and low SES (− 0.16), with treatment balanced (SMD 0.00). For follow-up dropout among baseline responders (completers versus dropouts), the profile differed: baseline PROM was the strongest correlate (SMD + 0.38), followed by comorbidity (− 0.36) and disease stage (− 0.28), and treatment was now imbalanced (SMD − 0.16), reflecting differential dropout in the treated group. Separating the mechanisms therefore revealed treatment-associated dropout that was not apparent in the pooled comparison, with direct implications for the weight specification in Step 2.

### Primary analyses

Table 3 summarizes results. The complete-case estimate was 1.21 PROM points (95% CI: 0.69–1.72). The IPW-adjusted estimate was 1.22 points (95% CI: 0.69–1.74, robust sandwich SE), indicating minimal residual bias from the dropout mechanism under MAR. The IPW confidence interval is marginally wider than the complete-case interval, consistent with the additional uncertainty introduced by weight estimation.

Two-stage inverse probability weighting was applied to operationalize both missingness mechanisms (Fig. 2). The baseline-response and follow-up-response models showed modest discrimination (AUC 0.62 and 0.67, respectively), and the two-stage weights were well-behaved (mean 2.1, maximum 12.0, coefficient of variation 0.35). The two-stage IPW estimate (1.23 PROM points; 95% CI: 0.67–1.79) corroborated both the complete-case (1.21; 0.69–1.72) and the single-stage IPW estimate (1.22; 0.69–1.74), with a marginally wider interval reflecting the additional baseline-response weighting step. This concordance is expected given that treatment was balanced with respect to baseline response.

Under the secondary scenario (true effect + 4.0 PROM points), the complete-case, single-stage IPW, and two-stage IPW estimates were 4.33 (3.82–4.85), 4.33 (3.81–4.84), and 4.31 (3.77–4.85), respectively. The tipping point was δ* = 30.5 points, with the 95% confidence interval first including zero at δ ≥ 26.3 points; the result was classified as stable under the illustrative thresholds (Table 3).

**Table 3:** Simulated worked example: treatment effect estimates across analytical strategies

Analysis/result	Primary scenario: true effect + 0.8	Secondary scenario: true effect + 4.0
N with complete follow-up PROM	3,829	3,882
Complete-case estimate	1.21 (0.69 to 1.72)	4.33 (3.82 to 4.85)
Single-stage IPW estimate	1.22 (0.69 to 1.74)	4.33 (3.81 to 4.84)
Two-stage IPW estimate	1.23 (0.67 to 1.79)	4.31 (3.77 to 4.85)
Tipping point δ*	7.2 points	30.5 points
95% CI first includes zero for δ ≥	4.7 points	26.3 points
Robustness classification	Sensitive	Stable

Treatment effect estimates across analytical strategies in the simulated registry-PROM cohort (*N* = 8,000). Effect estimates are PROM points with 95% confidence intervals. IPW = inverse probability weighting. The single-stage IPW model weights follow-up response among baseline responders. The two-stage IPW model combines a baseline-response weight and a follow-up-response weight. The tipping point δ* denotes the value of the asymmetric delta adjustment at which the point estimate crosses zero. The “95% CI first includes zero” row denotes the δ value at which the lower confidence bound first crosses zero. Robustness classification thresholds are illustrative: Stable (δ* > 10), Sensitive (δ* 5–10), and Unstable (δ* < 5), and should be adapted to the minimally important difference or meaningful score difference of the PROM instrument used

### MNAR sensitivity and tipping-point analysis

**Fig. 3:**
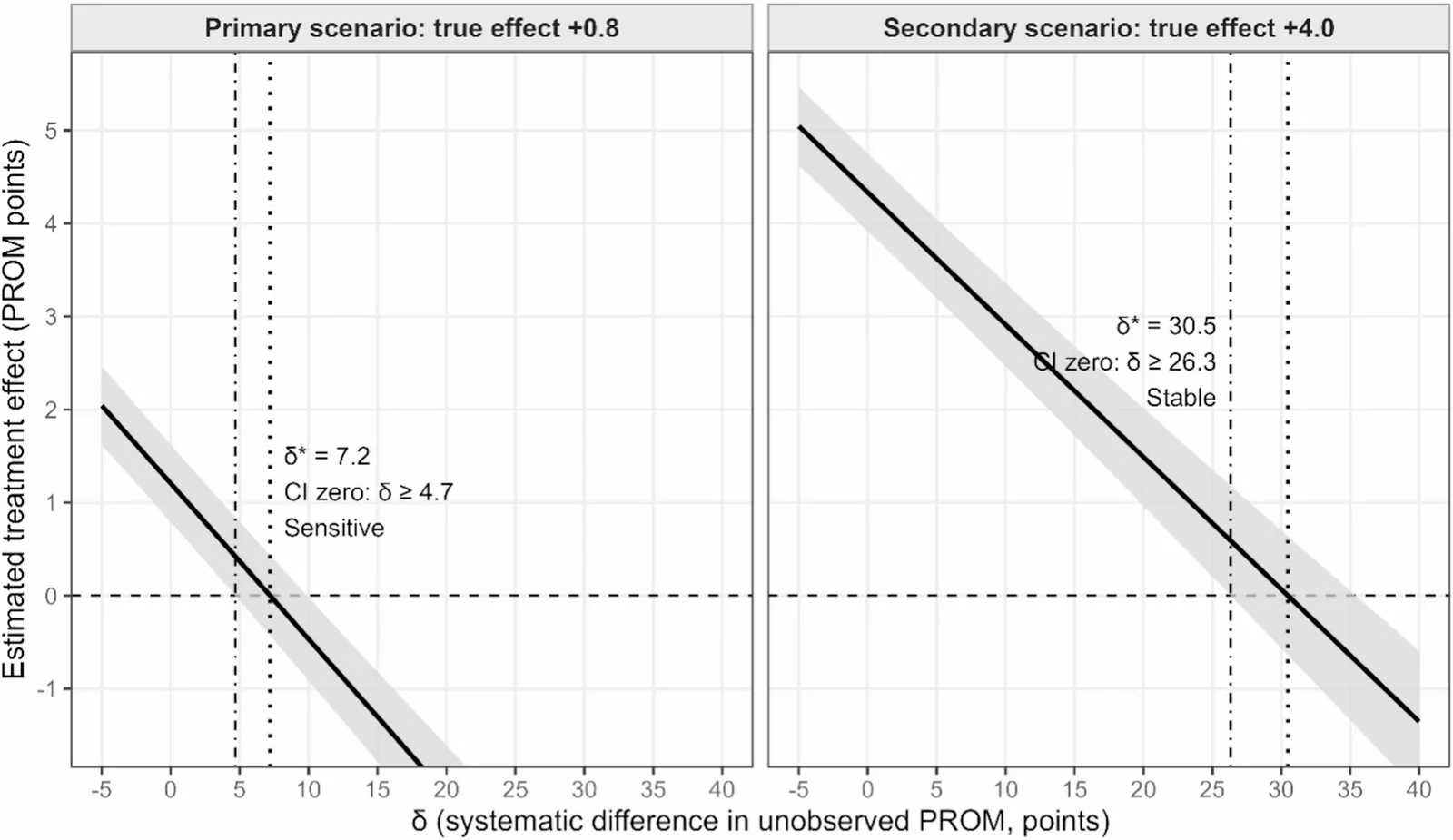
MNAR delta-adjustment sensitivity and tipping-point analysis. MNAR delta-adjustment sensitivity and tipping-point analysis in the primary and secondary simulated scenarios. Solid line: estimated treatment effect on follow-up PROM across values of δ, where δ represents the assumed systematic downward shift in unobserved PROM among treatment-group non-responders; control-group non-responders are shifted by δ/3. Shaded band: 95% confidence interval. Horizontal dashed line: null effect. Dotted vertical line: tipping point δ*, where the point estimate crosses zero. Dot-dash vertical line: δ value at which the 95% confidence interval first includes zero. In the primary scenario, δ* = 7.2 and the result is classified as sensitive; in the secondary scenario, δ* = 30.5 and the result is classified as stable. Simulated cohort, *N* = 8,000

Under the asymmetric delta-adjustment scenario, the estimated treatment effect decreased monotonically with increasing δ (Fig. 3). The point estimate crossed zero at δ* = 7.2 points (tipping point). Separately, the lower bound of the 95% confidence interval included zero from approximately δ ≥ 4.7 points onward, indicating that statistical significance at the conventional level would be lost before the point estimate itself reverses. The tipping point δ* refers specifically to the point estimate crossing zero; the CI threshold is a complementary measure of inferential fragility. Using the proposed robustness classification, this result is classified as sensitive under the illustrative thresholds: the primary conclusion is not overturned under modest MNAR assumptions, but would reverse if non-responders in the treatment group were systematically 7.2 PROM points worse than predicted under MAR. Given that observed non-response SMDs (0.16–0.27) corresponded to 4–6 PROM points between responders and non-responders, a residual unobserved difference of 7.2 points may be at the boundary of clinical plausibility, depending on context.

## Discussion

### Principal findings

A full-text methods-section audit of 61 registry-based observational PROM studies identified from four database searches (2018–2024; 17 Nordic, 44 global) reveals a consistent two-level gap in MNAR sensitivity practice across settings and registries. At the level of analytic completeness, tipping-point analysis was not reported in the accessible methods or supplementary material of any of the 61 included registry-PROM studies; delta adjustment reported in only one study (1.6%), applied as a sensitivity scenario rather than a primary analytic approach. At the level of basic missing-data transparency, 39% stated no strategy and only 28% distinguished never-responders from dropout.

The five formal MNAR analyses identified used methods that typically require specialist expertise and stronger modeling assumptions than are routinely feasible in many registry settings. These are rigorous approaches, but they are not suitable as a minimum standard for routine registry-PROM reporting. The workflow proposed here fills a different niche: not replacing rigorous MNAR modeling where warranted, but providing a feasible, standardizable sensitivity floor that every registry-PROM study can meet.

### Why the gap persists and why tipping-point analysis addresses it

The gap between MNAR awareness and MNAR practice likely persists because the available formal methods require specialist statistical expertise and stronger modeling assumptions than are routinely feasible in registry settings. The National Research Council recommended that sensitivity analyses for departures from MAR should be a standard component of clinical reporting [6], yet practical implementation tools tailored to registry settings have remained absent. Tipping-point analysis resolves this by reframing the question. Rather than “what is the MNAR mechanism?”, an unidentifiable question, it asks “how wrong would MAR need to be for our conclusion to change?” [5, 8] This is both answerable and clinically communicable, and requires no parametric commitment to a specific MNAR data-generating process.

### Interpretation of the worked example

The simulated worked example illustrates all three workflow steps. Non-response diagnostics confirmed meaningful selection (SMDs 0.16–0.27) consistent with published Nordic register data [3, 12]. Despite this, CC and IPW estimates were nearly identical, illustrating that IPW can corroborate CC inference when the response propensity is adequately modelled by observed baseline covariates. The similarity between CC and IPW estimates suggests that observed covariates capture key drivers of response, but does not constitute formal evidence for the MAR assumption, which remains fundamentally untestable from observed data alone.

The tipping-point result (δ* = 7.2 points; classified: sensitive) provides a concrete, communicable answer. The conclusion is not overturned under modest MNAR assumptions, but a residual differential of 7.2 points among unobserved treatment-group patients would reverse it. Whether this is clinically plausible is a domain question that the researcher and reader can now assess directly. To illustrate how robustness classification behaves when the underlying treatment effect is clinically meaningful, a secondary scenario with a true effect of + 4.0 PROM points was examined. In this scenario, the tipping point was δ* = 30.5 points (classified as stable under the illustrative thresholds), and the 95% confidence band included zero only for δ ≥ 26.3 points. A systematic MNAR deviation of this magnitude, non-responders in the treatment group being 30 points worse than predicted, is implausible in most clinical contexts, underscoring that the workflow produces appropriately stable classifications when evidence for treatment benefit is strong.

### Practical recommendations

Registry-PROM studies are encouraged to adopt the minimum reporting set in Table 1 as standard practice. The most impactful additions, often entirely absent in current practice, are: explicit separation of never-responders from dropout; SMD-based non-response diagnostics; and at least one MNAR tipping-point analysis. These require no new data collection, no specialist software, and can be implemented using the R script (provided as Simulation script.R).

### Limitations

The audit was conducted on full-text methods sections from a geographically and temporally bounded sample and reflects reporting practice rather than what analyses were necessarily performed: analyses may have been conducted without being described in the methods section, or reported only in supplementary material not retrieved by the search, and we did not contact authors to verify unreported analyses, so all findings are framed as ‘not reported’ rather than ‘not done’. The worked example uses an illustrative asymmetric δ specification; in applied analyses, the direction and relative magnitude of differential MNAR by group should be guided by subject-matter knowledge. The asymmetric δ specification is illustrative and the ratio should be adapted to clinical context. The robustness classification thresholds are illustrative and must be adapted to the established minimally important difference (MID) of the PROM instrument used; fixed numerical thresholds detached from instrument-specific MIDs risk misclassifying results. Confidence intervals in the tipping-point figure are model-based and should be interpreted as approximate. The workflow does not address missing covariate data.

## Conclusions

Tipping-point sensitivity analysis for MNAR data was not reported in the accessible methods or supplementary material of any of the 61 included registry-PROM studies; delta adjustment was reported in only one study as a sensitivity check. The formal MNAR methods that do appear require expertise beyond routine registry reporting. A pragmatic three-step workflow, non-response diagnostics, IPW-first primary analysis, and standardized tipping-point sensitivity, provides a feasible and clinically interpretable standard that every registry-PROM study can adopt. Adoption of the proposed minimum reporting set would represent a substantive and achievable improvement in field standards.

## Supplementary Information

Below is the link to the electronic supplementary material.

Supplementary Material 1

Supplementary Material 2

Supplementary Material 3

Supplementary Material 4

## Data Availability

The simulation R script is provided as a supplementary file (Simulation_script.R). Audit extraction data are presented in full in Supplementary Table S1. No individual patient data were used.

## Abbreviations

CC: Complete–case analysis
IPW: Inverse probability weighting
MAR: Missing at random
MCAR: Missing completely at random
MID: Minimal important difference
MNAR: Missing not at random
PROM: Patient–reported outcome measure(s)
SES: Socioeconomic status
SMD: Standardized mean difference
