# Supplementary material for: A pragmatic workflow for handling missing PROM data in registry studies: a methodological scoping review and simulated worked example
Source: BMC Med Res Methodol. 2026 Oct 19;26:225. doi: 10.1186/s12874-026-03058-x (PMC13682039; doi:10.1186/s12874-026-03058-x)
Supplement: Supplementary file 1 [file 12874_2026_3058_MOESM1_ESM.pdf]

Supplementary Table S1. Full extraction: all included studies. Nordic: 39 screened (PubMed) → 22 registry obs → 17 primary analytic sample. Global: 386 screened (Embase/CINAHL/PubMed global) → 44 included. Total primary analytic sample: n = 61.

*Registry obs = registry-based observational study. * Excluded from primary analytic sample (non-registry design). Indirect = mentioned in results without explicit methods discussion. MNAR informal = MNAR discussed without formal model. MNAR formal = explicit MNAR model. — = not applicable or not stated. Nordic sample: PubMed search 2026-03-05. Global sample: Embase, CINAHL, PubMed global search 2026-04-22.*

Study	Title (abbreviated)	Design	Register/source	PROM	Response rate reported	Missingness described	Missing strategy (fulltext)	Responder comparison	MNAR sensitivity	MNAR
NORDIC SAMPLE (PubMed Nordic search, n = 39 screened; 22 registry/routine obs; 17 primary analytic sample; * excluded from primary analytic sample)										
Kjærvik 2024	PROMs in hip fracture patients	Registry obs	NHFR	EQ-5D-3L	Yes	Yes	MI (FCS)	Yes – pattern	Formal	Shared parame joint mo
Mathew 2022	Enthesitis in PsA and axSpA (DANBIO)	Registry obs	DANBIO	SPARCC/PRO	Yes	Yes	Not stated	Yes – baseline	None	—
Burnand 2021	EQ-5D-3L by text vs paper in trauma setting	Other*	SA trauma	EQ-5D-3L	Yes	Yes	CC	Yes – baseline	None	—
Andersen 2019	HRQoL in primary antibody deficiency	Registry obs	CRD database	CVID_QoL/WHOQOL	No	Yes	Not stated	None	None	—
Kjaer 2018	Non-response at HRQoL follow-up in septic shock	Registry obs	Statistics Denmark	SF-36	Yes	Yes	Single imp/CC	Yes – baseline	Informal	'Not MC stated
Olsen 2021	HRQoL after critical illness (NONSEDA)	RCT*	NONSEDA	SF-36/Barthel	Yes	Yes	CC	None	None	—
Haukeland 2022	Validation of Leicester Cough Questionnaire in COPD	Validation*	Clinical KOL	LCQ	No	Yes	FIML	None	None	—
Liu 2023	Single-arm studies with PRO data in oncology: review	Review*	Literature	Various	Yes	Yes	Varies	Yes – notes	None	—
Mortensen 2024	HRQoL follow-up of the AID-ICU trial	RCT*	AID-ICU	EQ-5D-5L	Yes	Yes	MI	None	None	—
Nielsen 2020	Strategies to improve PRO completion rates	Registry obs	Danish MM registry	EORTC QLQ-C30	Yes	Yes	Mixed effects	Yes – baseline	Informal	MNAR mechar discuss
Nielsen 2024	Reasons for intermittent missing PRO data	Registry obs	QoL-MM	EORTC QLQ-C30	Yes	Yes	Regression (salvage)	Yes – early/late	Formal	MID-ba MNAR classifi
El-Hajj 2023	PROMs 1, 2, and 5 years after cervical spine surgery	Registry obs	Swespine	EQ-5D-3L/NDI	Yes	No	Mixed ANOVA	None	None	—
Lillevik 2018	Validation of CROQ-PCI	Validation*	UNN	CROQ-PCI	Yes	Yes	Not stated	None	None	—
Parai 2020	Patients lost to follow-up after lumbar spine surgery	Registry obs	Swespine	GA/ODI/VAS/EQ-5D	Yes	Yes	Regression pred.	Yes – baseline	Formal	Selecti model
Tolstrup 2018	Danish translation of patient feedback form	Validation*	Prostate cancer	Patient Feedback	Yes	Yes	EM imputation	None	None	—
Sand-Svartrud 2024	Completeness of repeated PROMs in rehabilitation	Registry obs	RehabNytte	EQ-5D-5L/PROMIS	Yes	Yes	Logistic regression	Yes – baseline	None	MAR/M mentio only

Study	Title (abbreviated)	Design	Register/source	PROM	Response rate reported	Missingness described	Missing strategy (fulltext)	Responder comparison	MNAR sensitivity	MNAR
Garratt 2018	Three PROMs following ankle fracture fixation	Registry obs	Ankle fracture cohort	SF-36/OMAS/SEFAS	Yes	Yes	Mean imputation	None	None	—
Mauland 2024	PROs after dental implant rehabilitation (NNIS)	Registry obs	NNIS	Satisfaction survey	Yes	Yes	Not stated	Yes – baseline	None	—
Leick 2022	Non-participation in prevention programme	Feasibility*	GP clinics	Lifestyle Qs	Yes	Yes	Not stated	Yes – baseline	None	—
Hernar 2021	PROMs in diabetes consultations (DiaPROM RCT)	RCT pilot*	DiaPROM	PAID/DDS/WHO-5	Yes	Yes	Person-mean subst.	Yes – baseline	None	—
Kaur 2023	Non-respondents in national spine registry	Registry obs	NORspine	ODI/NRS	Yes	Yes	Not stated	Yes – early/late	Informal	'Attritio was ra
Hjollund 2019	Fifteen years' use of PROMs	Registry obs	AmbuFlex/WestChronic	Multiple PROMs	Yes	Yes	Not stated	None	None	—
Rosing 2024	Tooth-loss related masticatory experiences	Cross-sectional*	Survey	Dental health	No	No	Not stated	None	None	—
Lyhne 2024	Missingness mechanisms in colorectal cancer PROMs	Registry obs	Danish Clinical Reg.	FCRI-SF/EQ-5D-5L	Yes	Yes	CC	Yes – early/late	Informal	MCAR plausib check
Balstad 2019	Patient interpretation of PG-SGA Short Form	Qualitative*	Cancer clinic	PG-SGA Short Form	No	Yes	Not stated	None	None	—
Grønne 2021	Cost-effectiveness of GLA:D programme	Registry obs	GLA:D	EQ-5D	Yes	Yes	MI (chained eq.)	Yes – CC sens.	Informal	MI und MAR; C sensitiv
Andersen 2020	HRQoL in porphyria cutanea tarda	Registry obs	Porphyria registry	SF-12	Yes	Yes	Not stated	None	None	Ethics preven analysi
Iversen 2019	Validity of APEQ-DC in childhood diabetes	Registry obs	Childhood Diabetes	APEQ-DC	Yes	Yes	Not stated	None	None	Missing analysi
Benestad 2019	HRQoL after obesity treatment RCT	RCT*	Camp treatment	KINDL/SF-36	Yes	Yes	MI (FCS)	None	None	—
Talvitie 2023	Missingness in EPIC-26	Survey/other*	Finland survey	EPIC-26	Yes	Yes	Logistic regression	Yes – baseline	Informal	'Requir careful conside
Tolstrup 2022	PROs during checkpoint inhibitor treatment	RCT*	PROMelanoma	EQ-5D-5L/FACT-M	Yes	Yes	Guideline imputation	None	None	—
Marthinsen 2022	Sleep patterns in parents of preterm infants	Feasibility*	Hospital wards	RAND-36	Yes	Yes	Not stated	Yes – baseline	None	—
Selinheimo 2023	RCT protocol for persistent physical symptoms	Protocol*	Finnish registers	15D questionnaire	No	Yes	FIML	Yes – baseline	None	—
Jansen 2024	Sex differences in mobility after hip fracture	Other*	Pooled data	DMO (digital)	No	Yes	Quantile regression	None	None	—
Heiberg 2018	QOLIBRI-overall scale	Validation*	UNN stroke	QOLIBRI-OS	Yes	Yes	Not stated	None	None	—

Study	Title (abbreviated)	Design	Register/source	PROM	Response rate reported	Missingness described	Missing strategy (fulltext)	Responder comparison	MNAR sensitivity	MNAR
	after stroke: validation									
**GLOBAL SAMPLE (Embase + CINAHL + PubMed global search, n = 44 included)**										
Cabitza 2019	PROs in the wild: validity in electronic registry	Registry obs	Datareg (IOG)	VAS pain, COMI, SF-12	Yes	Yes	Other (proxy analysis)	Yes – early/late	Informal	—
Cöster 2023	EQ-5D data and follow-up rate in orthopaedic registers	Registry obs	Swespine, SHR, SKAR	EQ-5D, EQ-VAS	Yes	Indirect	CC	Yes – baseline	None	—
Hareni 2023	Lumbar spinal stenosis outcomes (Swespine)	Registry obs	Swespine	NRS pain, ODI	Yes	No	CC	None	None	—
van Dooren 2023	Improvement after THA (LROI)	Registry obs	LROI	NRS, EQ-5D, HOOS-PS, OHS	Yes	Yes	CC	None	Informal	MAR assump stated
Torisho 2019	Minor influence of patient education on THR outcomes	Registry obs	SHR	EQ-5D, pain VAS	Yes	Indirect	CC	Yes – baseline	None	—
Hüsken 2024	PROMs according to prior pelvic osteotomy	Registry obs	LROI	EQ-5D, OHS, HOOS-PS	Yes	No	Not stated	None	None	—
Jännebring 2024	Patient-reported salpingitis on hysterectomy outcome	Registry obs	GynOp	Satisfaction, pain	Yes	No	CC	None	None	—
Perrier 2022	Physical activity trajectories and melanoma risk	Registry obs	NOWAC	PA questionnaire	Yes	No	MI (latent class mixed model)	None	None	—
Mullins 2019	Sexual QoL after radical prostatectomy	Registry obs	NC Central Cancer Registry	PCSI	No	No	MI (FCS)	None	None	—
Adam 2020	HRQoL in long-term prostate cancer survivors	Registry obs	NICER	EORTC QLQ-C30/PR25	Yes	Yes	MI – MICE	Yes – baseline	None	—
Deng 2024	Fatigue and psychological symptoms in AYA cancer survivors	Registry obs	Cancer Registry of Norway	PHQ-9, fatigue	Yes	Indirect	Model-based (LPA)	None	Informal	—
van Egmond 2020	Three distinct recovery patterns after TKA	Registry obs	LROI	OKS, NRS, EQ-5D	Indirect	No	CC	Yes – other	None	—
Zhang 2024	Dupilumab effectiveness in atopic dermatitis (BioDay)	Registry obs	BioDay registry	POEM, DLQI, NRS	No	No	MI	None	None	—
Zhou 2023	Joint replacement PROMs in SMART registry	Registry obs	SMART registry	VR12, WOMAC	Yes	Yes	Model-based (mixed models)	Yes – early/late	Informal	Pattern mixture
Singh 2023	Cervical spine fracture outcomes (Swespine)	Registry obs	Swespine	EQ-5D-3L, NDI	Yes	Indirect	CC	Yes – baseline	Informal	Attrition check

Study	Title (abbreviated)	Design	Register/source	PROM	Response rate reported	Missingness described	Missing strategy (fulltext)	Responder comparison	MNAR sensitivity	MNAR
Younossi 2021	PROMs in NAFLD (GNR)	Registry obs	GNR	CLDQ-NASH, FACIT-F	No	No	Not stated	None	None	—
SOReg 2022	HRQoL over time after bariatric surgery	Registry obs	SOReg	HRQoL (not specified)	Indirect	Indirect	CC	Yes – baseline	None	—
Guo 2022	Rates of diagnosis and treatment for cystic fibrosis	Registry obs	CF registries	Survey data	Yes	Indirect	Other (extrapolation)	None	None	—
Babington 2017	PROM utility for predicting spinal surgery	Registry obs	Virginia Mason Spine Clinic	PHQ-8, ODI, PROMIS	Yes	No	CC	None	None	—
Ingelsrud 2022	International hip and knee arthroplasty registries	Registry obs	13 registries	EQ-5D, OKS, OHS	Yes	Yes	Other (standardisation)	Yes – early/late	None	—
Kongsted 2020	Danish Chiropractic LBP Cohort (ChiCo)	Registry obs	ChiCo	RMDQ, STarT	Yes	No	MI – chained equations	Yes – baseline	None	—
Poll-Franse 2022	PROFILES registry: cancer survivorship PROMs	Registry obs	PROFILES	EORTC QLQ-C30	Yes	Indirect	Model-based (dimension reduction)	Yes – early/late	None	—
Hoskins 2024	Revision rates vs PROMs (AOANJRR)	Registry obs	AOANJRR	OHS, OKS, EQ-5D	Yes	Yes	CC (vs MI, vs delta adj.)	None	Formal	Delta adjustm
Sinnige 2024	Personalised outcomes forecasts in chronic care	Registry obs	Chronic CareNet	HRQoL, FWD	Yes	Yes	CC	Yes – early/late	None	—
Outpatient psych 2023	ML prediction of non-improvement in mental healthcare	Registry obs	Psych register	OQ-45, MHC-SF	Indirect	No	CC	None	None	—
Gyllensten 2018	Costs and HRQoL in MS (PAR/SMSreg)	Registry obs	PAR, SMSreg	EQ-5D, EQ-VAS	Yes	No	Single imputation (LOCF-like)	Yes – baseline	None	—
Sun 2022	Sequential MI for HRQoL missing data after bariatric surgery	Registry obs	SOReg	SF-36, SF-6D	Yes	Yes	MI – sequential MICE/PMM	Yes – baseline	Informal	MNAR limitatio
Katayama 2023	Alcohol and fatigue in SLE (Lupus registry)	Registry obs	Lupus registry	Fatigue scale	No	Yes	MI – MICE	None	None	—
Purvis 2022	Linking stroke registry and hospital resource data	Registry obs	AuSCR	EQ-5D, EQ-VAS	Yes	No	Not stated	None	None	—
Kalweit 2021	ML-based prediction in RA registry (SCQM)	Registry obs	SCQM	SF-12, DAS28	Indirect	No	Not stated (ML/LSTM)	None	None	—
Bariatric 2021	Loss to follow-up after bariatric surgery	Registry obs	Bariatric DB (US)	PROMIS-10, GERD	Yes	Yes	CC	Yes – baseline	None	—
Fry 2024	Outcomes of ventral hernia repair (MSQC-COHR)	Registry obs	MSQC-COHR	PROMIS	Yes	Indirect	IPW (non-response weights)	None	None	—
Wide 2024	HRQoL in young adult cancer survivors (Sweden)	Registry obs	Swedish cancer registries	EORTC QLQ-C30	Yes	Indirect	CC	Yes – baseline	None	—

Study	Title (abbreviated)	Design	Register/source	PROM	Response rate reported	Missingness described	Missing strategy (fulltext)	Responder comparison	MNAR sensitivity	MNAR
Mannion 2023	Evaluate attrition in spine registry	Registry obs	Schulthess Klinik	COMI, GTO	Yes	Yes	MI – other (prediction)	Yes – early/late	Informal	Pattern mixture (mentio
Ingebrigtsen 2022	Non-respondents do not bias outcome after cervical spine surgery	Registry obs	NORspine	NRS, EQ-5D-3L, GPE	Yes	Yes	Other (telephone interviews)	Yes – baseline	Informal	—
Rojas-Gualdrón 2024	HRQoL loss in seropositive RA (ARTMEDICA)	Registry obs	ARTMEDICA, Colombia	EQ-5D-3L	Yes	No	CC	None	None	—
de Rooij 2018	Cancer survivors not participating in PROFILES	Registry obs	PROFILES / NCR	EORTC QLQ-C30	Yes	Yes	Other (hot-deck comparison)	Yes – baseline	None	—
van der Vliet 2019	Longer-term PROMs in Dutch trauma registry (DNTD)	Registry obs	DNTD	EQ-5D-3L, EQ-VAS	Yes	Yes	Other (telephone follow-up)	Yes – baseline	None	—
Athanassiou 2021	Golimumab on work productivity and QoL in axSpA/PsA	Registry obs	Greek NIS registry	EQ-5D-5L, WPAI-SHP	Yes	No	CC	None	None	—
Ramsey 2019	Cancer survivors in PROFILES: participation and HRQoL	Registry obs	PROFILES	EORTC QLQ-C30, HADS	Yes	Yes	Model-based (linear mixed models)	Yes – baseline	None	—
Lyhne 2024 (global)	Missingness and generalizability in colorectal cancer PROMs	Registry obs	DCCG / RKKP	FCRI-SF, EQ-5D-5L	Yes	Yes	Other (early vs late responders)	Yes – baseline	None	—
Kaur 2023 (global)	Non-respondents in national spine surgery registry	Registry obs	NORspine	ODI, NRS, EQ-5D, GPE	Yes	Yes	Other (extra non-responder study)	Yes – baseline	None	—
Ayilara 2019	Impact of missing data on PROM change estimates	Registry obs	WRHA Joint Replacement Registry	SF-12v2	Yes	Yes	Model-based (mixed models)	Yes – baseline	Formal	Selecti model
Rossen 2024	Electronic PROMs in cancer rehabilitation	Registry obs	Municipal registers (Copenhagen)	FACT-G	Yes	Yes	CC	Yes – baseline	None	—
Steinberg 2020	Large improvements in HRQoL in AF patients (ORBIT-AF)	Registry obs	ORBIT-AF registry	AFEQT	Yes	No	Single imputation (MCMC)	None	None	—
Jayakumar 2024	Mental health constructs in hip/knee OA	Registry obs	Joint Health Program	KOOS JR, HOOS JR, OSPRO-YF	Yes	No	CC	None	None	—
Buckell 2024	Effect of bariatric surgery on HRQoL (UK NBSR)	Registry obs	UK NBSR	EQ-5D-5L	Yes	No	CC	None	None	—
Andersen 2020 (global)	HRQoL in porphyria cutanea tarda (Norwegian registry)	Registry obs	Norwegian Porphyria Registry	SF-12	Yes	Indirect	CC	None	None	—
