# Supplementary material for: A pragmatic workflow for handling missing PROM data in registry studies: a methodological scoping review and simulated worked example
Source: BMC Med Res Methodol. 2026 Oct 19;26:225. doi: 10.1186/s12874-026-03058-x (PMC13682039; doi:10.1186/s12874-026-03058-x)
Supplement: Supplementary file 3 [file 12874_2026_3058_MOESM4_ESM.pdf]

Minimum Reporting Set for Registry-Based PROM Studies with Missing Data

A checklist for authors and reviewers. Companion to the three-step workflow (non-response diagnostics → IPW-first analysis → MNAR tipping-point sensitivity).

Done	Domain	Minimum requirement
☐	**PROM flow**	Collection timepoints stated; mode of data collection (paper / electronic / mixed); number invited per timepoint.<br>*Enables identification of structural missingness patterns.*
☐	**Missingness profile**	Proportion missing per timepoint reported; never-responders distinguished from follow-up dropout.<br>*Missingness type determines the appropriate analytic strategy.*
☐	**Non-response diagnostics**	Responder vs. non-responder comparison on ≥5 prespecified baseline covariates (age, comorbidity, disease severity, socioeconomic factors, treatment) using standardized mean differences (SMD); baseline non-response and follow-up dropout assessed separately.<br>*Quantifies potential for selection bias; SMD is preferred over p-values.*
☐	**Primary strategy**	Method stated (complete-case / IPW / multiple imputation); response-model specification and predictors reported; weight diagnostics provided if IPW (e.g. weight distribution, truncation).<br>*Supports reproducibility and critical appraisal of the MAR assumption.*
☐	**Results side-by-side**	Complete-case and primary-strategy estimates reported together.<br>*Transparent comparison; highlights sensitivity to the choice of strategy.*
☐	**MNAR sensitivity**	At least one delta-adjustment / tipping-point analysis: range of δ tested and the tipping point δ^* at which the substantive conclusion changes.<br>*Quantifies how severe an MNAR departure would need to be to change inference.*
☐	**Robustness classification**	Result classified as Stable ($\delta^* > 10$) / Sensitive (δ^* 5–10) / Unstable ($\delta^* < 5$), with thresholds adapted to the instrument scale.<br>*Provides a clinically interpretable summary of MNAR sensitivity.*

Threshold values for the robustness classification are indicative and should be adapted to the scale and minimally important difference (MID) of the PROM instrument used. CC = complete-case analysis; IPW = inverse probability weighting; MNAR = missing not at random; SMD = standardized mean difference.
